# Towards a better understanding of antimicrobial resistance dissemination: what can be learnt from studying model conjugative plasmids?

**DOI:** 10.1186/s40779-021-00362-z

**Published:** 2022-01-10

**Authors:** Zhen Shen, Christoph M. Tang, Guang-Yu Liu

**Affiliations:** 1 Sir William Dunn School of Pathology, University of Oxford, Oxford, OX1 3RE UK; 2 Department of Laboratory Medicine, Ren Ji Hospital, School of Medicine, Shanghai Jiao Tong University, Shanghai, 200127 China

**Keywords:** Horizontal gene transfer, Antimicrobial resistance, Conjugative plasmids, Type IV secretion system, Restriction-modification systems, SOS response, Entry exclusion, Fertility inhibition

## Abstract

Bacteria can evolve rapidly by acquiring new traits such as virulence, metabolic properties, and most importantly, antimicrobial resistance, through horizontal gene transfer (HGT). Multidrug resistance in bacteria, especially in Gram-negative organisms, has become a global public health threat often through the spread of mobile genetic elements. Conjugation represents a major form of HGT and involves the transfer of DNA from a donor bacterium to a recipient by direct contact. Conjugative plasmids, a major vehicle for the dissemination of antimicrobial resistance, are selfish elements capable of mediating their own transmission through conjugation. To spread to and survive in a new bacterial host, conjugative plasmids have evolved mechanisms to circumvent both host defense systems and compete with co-resident plasmids. Such mechanisms have mostly been studied in model plasmids such as the F plasmid, rather than in conjugative plasmids that confer antimicrobial resistance (AMR) in important human pathogens. A better understanding of these mechanisms is crucial for predicting the flow of antimicrobial resistance-conferring conjugative plasmids among bacterial populations and guiding the rational design of strategies to halt the spread of antimicrobial resistance. Here, we review mechanisms employed by conjugative plasmids that promote their transmission and establishment in Gram-negative bacteria, by following the life cycle of conjugative plasmids.

Horizontal gene transfer (HGT) is defined as the intragenerational sharing of genetic material. HGT represents one of the most important evolutionary forces in bacteria as it enables rapid adaptation to changing environmental pressures through the acquisition of new traits such as virulence, metabolic pathways, and antimicrobial resistance (AMR) encoded by mobile genetic elements (MGEs) [1–5].

Among the MGE-conferred traits, AMR is of particular importance as it has been declared by WHO as one of the top 10 global public health threats facing humanity [6]. According to the O’Neill report, AMR accounts for at least 700,000 deaths globally every year and this figure is projected to reach 10 million by 2050. Moreover, the lost global production due to AMR between now and 2050 is predicted to be 100 trillion USD if no action is taken [7]. Nosocomial infections are a major concern posed by AMR in which patients with compromised immune systems are extremely vulnerable to bacterial infection [8]. Although most bacterial pathogens remain susceptible to the majority of clinically used antimicrobial agents such as carbapenems and amikacin, a few of them have developed extensive resistance to the action of antibiotics. Among these multi-resistant bacteria, “the ESKAPE pathogens”, which consist of *Enterococcus faecium*, *Staphylococcus aureus*, *Klebsiella pneumoniae (K. pneumoniae)*, *Acinetobacter baumannii (A. baumannii)*, *Pseudomonas aeruginosa (P. aeruginosa)*, and *Enterobacter* species, constitute the biggest threat to human health as they are responsible for the majority of nosocomial infections [8, 9].

Mechanistically, many ESKAPE pathogens, especially *K. pneumoniae* and *Enterobacter* species, acquire AMR mainly through the process of conjugation, which involves the concerted action of a mating pair formation (MPF) system and a matching DNA processing system, ultimately resulting in the transfer of one strand of DNA (the T-strand) from a donor cell to a recipient in which the complementary DNA strand is synthesized subsequently [10–12].

The most concerning resistance genes in clinical practice, such as *mcr-1*, *bla*_KPC_ and *bla*_NDM_, are all encoded by conjugative plasmids (CPs). CPs encode all genes required for their own transfer through mating and are particularly important for human and animal health, given their potential as vehicles to spread AMR [3]. To date, *mcr-1*, *bla*_KPC_ and *bla*_NDM_ have been identified in numerous countries across five continents [13–15]. MCR-1 belongs to the phosphoethanolamine transferase family and can help to modify the structure of lipid A, thus conferring resistance to colistin [16]. It is usually harbored by the epidemic IncI2 and IncX4 CPs and has mainly been identified in *Escherichia coli* (*E. coli*) [17]. KPC and NDM are the most prevalent carbapenemases and could hydrolyze almost all beta-lactams; *bla*_KPC_ and *bla*_NDM_, genes encoding KPC and NDM, are frequently detected in *K. pneumoniae* and *E. coli* and are mainly harbored by IncFII, IncFII/IncR, IncF and IncX3 CPs [14, 15].

Despite their clinical significance, how AMR-harboring CPs mediate their conjugative transfer and establish themselves in new bacterial hosts remain largely unexplored, especially for those epidemic IncI2, IncX3 and IncX4 CPs. Of note, AMR-harboring CPs from the IncF and IncFII families encode conjugative machinery and accessory proteins which are closely related to the well-studied model CPs, such as the F, R1 and the R100 plasmid [18, 19]. Therefore, here we summarize the molecular mechanisms by which CP-borne elements from Gram-negative bacteria facilitate conjugative transfer and become established in their new host, mainly using model CPs as examples. This review also attempts to shed light on the dissemination of AMR as the MPF systems of certain families of AMR-harboring CPs, such as the carbapenemase-encoding IncF and IncFII CPs, are highly homologous to those from model CPs such as the F and the R1 plasmid. However, extrapolating knowledge gained from studying model CPs to clinical AMR-harboring plasmids has a limit as the latter belong to a wide range of incompatibility groups, each of which may employ different strategies for host colonization and establishment. Proteins that are directly involved in MPF and conjugative DNA processing are not discussed here as they are reviewed in detail elsewhere [20–24]. For the convenience of discussion, we split the life cycle of CPs into three steps: before, during, and after conjugation.

## Before conjugation

CPs possess transfer genes that encode all factors necessary for the assembly of the MPF system including the conjugative pilus and the Type IV Secretion System (T4SS), as well as the relaxosome components required for the processing of plasmid DNA prior to transfer. The transfer genes are usually clustered in the *tra* region of CPs, and expression of *tra* region can impose significant fitness costs on the host bacterium [25]. Therefore, the expression of *tra* genes on CPs is tightly regulated. For instance, in F-like plasmids, the *tra* operon is normally repressed [26]. P_Y_, the primary promoter of the *tra* operon is under the direct control of a transcription activator, TraJ [27, 28]; TraJ is post-transcriptionally repressed by the fertility inhibition system FinOP [29]. FinP is an antisense RNA that binds to the 5’ untranslated region (UTR) of *traJ* mRNA, masking the ribosome binding site and inhibiting TraJ translation [30, 31]. FinO is an RNA chaperone that protects the FinP antisense RNA from RNase E degradation, thereby stabilizing the FinP-*traJ* mRNA duplex [29, 32, 33]. The *finOP* has also been spotted on the epidemic IncFII/IncR and IncFII KPC plasmid, which shares 95% sequence similarity with the *finOP* on the R1 plasmid [18], suggesting that it might also be involved in the regulation of the *tra* operon.

Conjugation imposes significant stress on the donor. Expression and assembly of a functional MPF system, such as the sex pilus from the F plasmid, triggers the *cpxAR* and σ^E^ stress response pathways in *E. coli* and leads to sensitivity to bile salts [34, 35]. To alleviate the fitness costs in donor cells during conjugation, TraR, a transcription factor encoded on the *tra* operon on the F plasmid, directly activates the σ^E^ extracytoplasmic stress pathway independent of the DegS/RseA signal transduction cascade, upregulating periplasmic proteases and chaperones to relieve the periplasmic stress following biosynthesis of the sex pilus [36].

## During conjugation

### Early expression of leading region genes

**Fig. 1:**
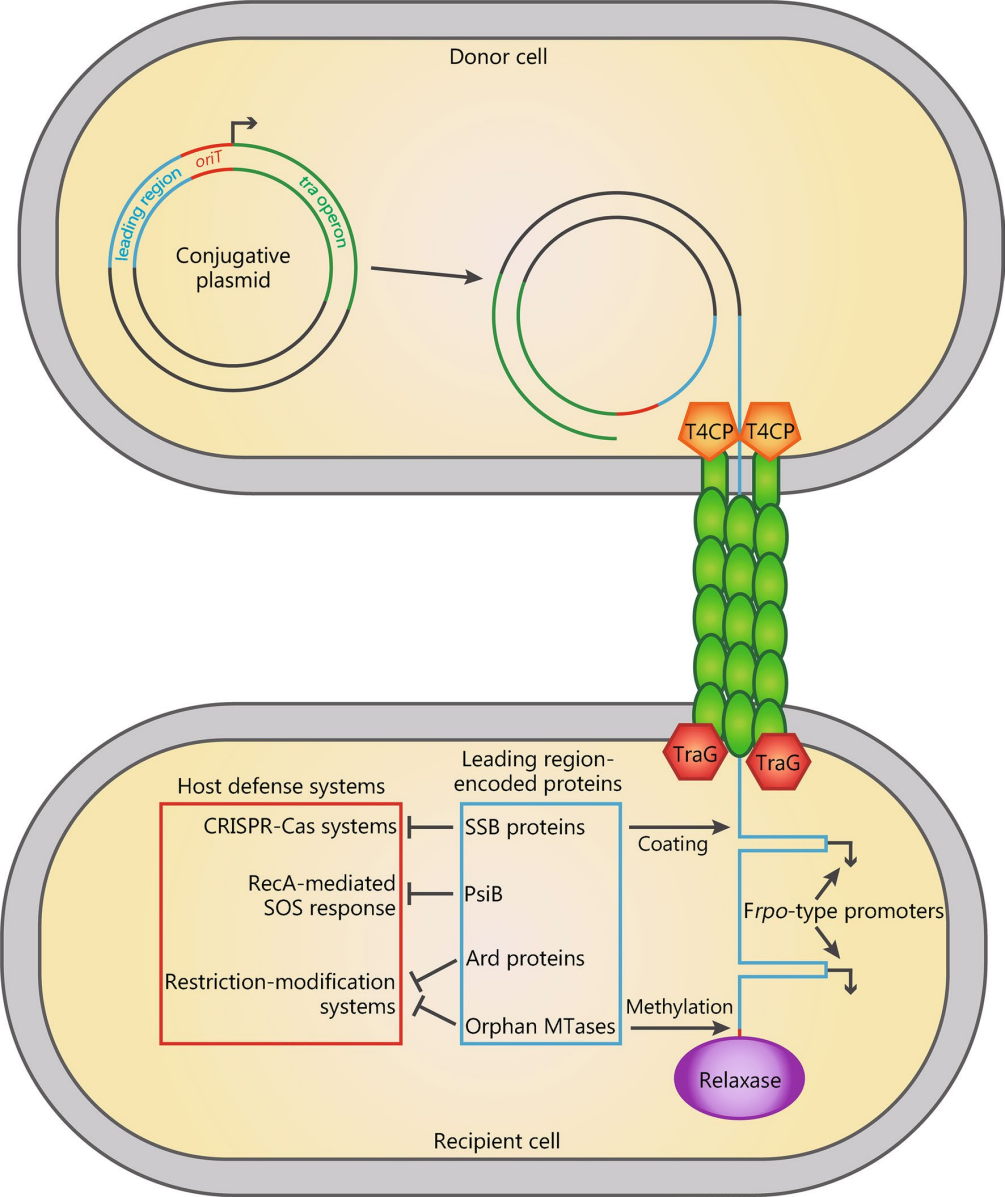
Conjugative plasmids employ proteins encoded on the leading region to antagonize defense systems in the recipient cell. The backbone of a generic CP is composed of the *tra* region that encodes all proteins necessary in conjugal transfer (green); the origin of transfer *oriT* (red); the leading region (blue), which is the first part of the CP transferred to the recipient cell coupled to the relaxase (purple) in a ssDNA form. F*rpo*-type promoters present on the leading region, when in ssDNA form, transiently switches on expression of genes encoding proteins (blue box) necessary to thwart host defense systems against foreign DNA (red box) in the recipient cell. Cas CRISPR-associated, CP conjugative plasmid, CRISPR clustered regularly interspaced short palindromic repeats, SSB single-stranded DNA binding, T4CP type IV coupling protein

Conjugative DNA transfer is initiated at a specific sequence on a CP, the *oriT*, which is recognized and nicked by the relaxase. The leading region of CPs is the sequence adjacent to the *oriT* and hence is the first to be transferred into the recipient cell (Fig. 1) [37, 38]. The leading region encodes proteins that play important roles in facilitating conjugative transfer. For instance, the leading region of the F and the R1 plasmid is a highly conserved 13–16 kb region that encodes for at least eight proteins [18, 38]. A conserved leading region has also been spotted on the epidemic IncFII/IncR and IncFII KPC plasmid, which is closely related to the R1 plasmid and shares about 90% sequence similarity [18]. Besides, for IncFII/IncR plasmid, it also has another leading region with IncR origin. How these two leading regions work together to facilitate conjugative transfer still needs further investigation.

Leading region genes can be expressed rapidly and transiently following conjugation from the partially transferred single stranded plasmid DNA in the recipient cell before its conversion into dsDNA, a phenomenon termed zygotic induction [39]. Mechanistically, zygotic induction requires F*rpo-*type promoters for single-strand initiation of DNA replication [40]. The F plasmid F*rpo* is a 328 bp sequence that adopts a stem-loop structure containing the -10 and -35 boxes required for binding of RNA polymerase and the synthesis of RNA primers [38]. Consistent with this, expression of leading region genes was shown to be driven by F*rpo*-like sequences in vivo [37, 41]. Furthermore, leading region genes whose early expression requires F*rpo*-like sequences are widespread in conjugative plasmids; their roles in promoting successful conjugation are discussed below.

### ssDNA binding proteins

Bacterial single-stranded DNA (ssDNA) binding (SSB) proteins typically consist of two domains: a ssDNA-binding oligonucleotide/oligosaccharide-binding (OB) domain and a structurally dynamic amphipathic C-terminal tail (SSB-Ct) [42, 43]. SSB proteins oligomerize through their OB domains to become active [42, 44]. For instance, each *E. coli* SSB protein has a single OB domain and becomes functional as a homotetramer [42–45]. SSB proteins diffuse on ssDNA as a sliding platform via reptation and form liquid–liquid phase separated condensates [46, 47]. SSB proteins are more than mere ssDNA binders, and act as bridges to recruit a repertoire of proteins to ssDNA through the SSB-Ct [45, 48]. These proteins are involved in various aspects of DNA metabolism including replication, repair, and recombination [49–51].

In the context of conjugation, upon entry of the plasmid into the recipient cell, ssDNA needs to be rapidly coated with SSB proteins. Although the recipient cell encodes its own SSB protein, conjugative plasmids frequently encode their own SSB in the leading region to ensure rapid and abundant expression, allowing protection of ssDNA from enzymatic degradation and facilitating conversion of the T-strand into dsDNA [52].

SSB proteins can also be part of the arsenal of CPs to subvert host defense. In a recent study using Tn-seq, Roy et al., identified an *hde* (host defense evasion) locus critical for the multidrug resistance-conferring IncC plasmid pVCR94 [53]. This locus enables the plasmid to paratisize *Vibrio cholerae* possessing a heterologous Type I CRISPR-Cas locus from *E. coli*. The *hde* locus contains five ORFs (*vcrx089–vcrx093*), with *vcrx089* and *vcrx090* encoding proteins that increase resistance against type I restriction-modification (R-M) systems, while *vcrx091*, *vcrx091*, and *vcxr093* encode an SSB, a single-strand annealing recombinase, and double-strand exonuclease related to Redβ and λExo of bacteriophage λ, respectively. These three proteins work in concert to repair CRISPR-Cas-mediated DNA double-strand breaks via homologous recombination between short sequence repeats in the plasmid [53].

### PsiB

During conjugation, plasmids enter the recipient cell in the form of ssDNA which induces an SOS response in *E. coli* [54–56]. Mechanistically, binding of RecA to single-stranded plasmid DNA leads to the formation of a RecA nucleofilament that catalyzes the auto-proteolytic cleavage of the LexA repressor. Cleavage of LexA de-represses genes belonging to the SOS regulon, which comprises around 40 genes in *E. coli* [55, 57]. Among the genes induced by the SOS response, several impede the conjugative transfer of plasmids. For instance, the division inhibitor SulA causes growth arrest, cell filamentation, and even cell death [58], leading to abortive CP transfer. Moreover, components that constitute DNA polymerase IV and V could introduce mutations into the transferred ssDNA [55]. To counteract the detrimental effects of the SOS response, conjugative plasmids belonging to different incompatibility groups, including the F plasmid, encode PsiB (plasmid SOS inhibition) [59]. Residing on the leading region, *psiB* expression also is subject to zygotic induction in IncF and IncI CPs [41, 60]. In addition to that, PsiB from IncFV plasmid pED208, along with SSB, was shown to be translocated directly through T4SS to suppress SOS response in the recipient [61]. PsiB binds directly to RecA, preventing nucleoprotein filament formation on ssDNA; this effect is more pronounced when ssDNA is bound by SSB such as during conjugal transfer [62]. PsiB-RecA interaction also prevents LexA autocleavage and hence the SOS response to ssDNA [62]. As PsiB-mediated RecA inhibition is based on PsiB-RecA binding, the inhibitory effect of PsiB varies in a species-specific manner [54].

### Anti-restriction-modification system proteins

Bacterial restriction-modification (R-M) systems are prokaryotic ‘innate immune systems’ that degrade exogenous DNA, in the form of phages and plasmids, entering the cell. R-M systems are typically comprised of a restriction endonuclease (REase) and a cognate methyltransferase (MTase). REases recognize and cleave unmethylated dsDNA of a specific sequence, whilst their cognate MTases protect DNA from REase cleavage by methylating adenine or cytosine residues within the recognized sequence. Differences in methylation allow R-M systems to discriminate invading DNA from host DNA to achieve targeted degradation [63–66].

As R-M systems act as a barrier against HGT, CPs have evolved several strategies to avoid restriction upon entry into recipient cells [66, 67]. The first plasmid anti-restriction system ArdA (alleviation of restriction of DNA) system was discovered in the IncI1 plasmid ColIbP-9 [68]. Of note, similar to PsiB, ArdA is encoded on the leading region of ColIbP-9 and is expressed by a F*rpo*-like promoter during conjugation before the incoming T-stand is converted into dsDNA [41].

ArdA proteins largely act on the REase of type I R-M systems but leave their modification activity largely unperturbed [69]. The crystal structure of ArdA reveals that ArdA dimers adopt an elongated curved cylindrical structure with regularly spaced helical grooves [70]. The surface of the ArdA dimer has a helical distribution of aspartate and glutamate residues, with the protein mimicking a 42-bp sequence of B-form DNA [70]. Later, a Cryo-EM structure of the type I R-M system EcoR124I complex demonstrated that type I R-M systems coordinate and regulate their endonuclease, methyltransferase, and translocase activities in a single complex by transitioning between different structural conformations [71]. Moreover, a structure of the EcoR124I in complex with ArdA reveals that ArdA does indeed bind DNA as a dimer, and intriguingly ArdA inhibits the function of the R-M complex without blocking its conformational transitions [71].

ArdB belongs to another family of proteins that antagonize type I R-M systems and was first identified from the IncN group conjugative plasmid pKM101 [72]. ArdB and ArdA are encoded on the leading region of pKM101 and their expression was controlled by ArdK and ArdR, two regulatory proteins encoded by the leading region [72]. Similar to ArdA, ArdB and its homologue KlcA specifically inhibit type I restriction in vivo, even though they have no activity against type I REase EcoKI in vitro [72, 73], indicating that the mechanism of REase inhibition that ArdB employs is unlikely to be direct, unlike ArdA [71, 73]. Consistent with this, the nuclear magnetic resonance (NMR) structure of KlcA from pBP136, an IncP-1b plasmid from *Bordetella pertussis*, demonstrated that KlcA has a novel fold, which does not appear to be a DNA mimic [73, 74]. Plasmids carrying *bla*_KPC-2_ are the primary cause of carbapenem resistance in *K. pneumoniae*, and often harbor *klcA*_HS_, a gene that encodes an ArdB homologue [75, 76]. Moreover, KlcA_HS_ was shown to increase transformation efficiency in *E. coli* encoding type I R-M systems belonging to different subclasses, suggesting KlcA_HS_ plays an important role in the dissemination of carbapenem resistance in *K. pneumoniae* [75, 76].

ArdC, the third type of restriction alleviation protein, was first identified in the IncW plasmid pSa and later also studied in another IncW plasmid R388 [77, 78]. ArdC is required for the conjugative transfer of R388 from *E. coli* to *Pseudomonas putida (P. putida)* by targeting a type I R-M system in the recipient, extending the broad host range of R388 [78]. ArdC shares low identity with ArdA and ArdB, but it has 38% identity with the N-terminal portion of TraC1 primase encoded by the IncP plasmid RP4 [77]. During conjugation, TraC1 is co-transferred with RP4 into the recipient cell, where it acts to generate RNA primers to initiate complementary strand synthesis of the T-strand [79]. Since the N-terminal portion of TraC1 is necessary for the conjugative transfer of TraC1, ArdC was also proposed to be transferred into the recipient and protect the T-strand [77]. The crystal structure of ArdC reveals that it consists of an N-terminal ssDNA-binding domain and a C-terminal metalloprotease domain [78]. Intriguingly, the metalloprotease activity of ArdC is dispensable for R388 conjugation into *P. putida* with the substrate of the metalloprotease domain unknown [78].

In contrast to R-M systems that consist of a coupled MTase and REase, many MTases lack cognate REase partners and are hence known as orphan MTases [80, 81]. The first genome-wide methylome study in bacteria by single-molecule real-time (SMRT) sequencing identified M.EcoGIX, an orphan MTase encoded on a conjugative plasmid pESBL in *E. coli* O104:H4 [82]. M.EcoGIX modifies adenine in a wide range of sequence contexts, and more surprisingly, it methylates only one strand of dsDNA [82]. Later, M.EcoGIX was shown to protect pESBL from restriction by R-M systems in a range of *E. coli* hosts during conjugation [83]. M.EcoGIX homologues are present on conjugative plasmids belonging to different incompatibility groups [83–85], in which M.EcoGIX encoded on pESBL from *E. coli* O104:H4 and M.BceJIII encoded on pBCG2315 of *Burkholderia cenocepacia* J2315 were characterized by Formenkov et al. [86]*.* It was shown that both MTases are encoded on the leading region and expressed early during conjugation, and they work in concert with DNA polymerase I to methylate the T-strand, protecting the plasmid from R-M systems during the conversion of the T-strand into DNA duplex [86].

## After conjugation

Successful conjugation does not guarantee the successful establishment and long-term persistence of CPs in their new hosts. Although many CPs encode beneficial traits such as AMR, carriage of CPs imposes a fitness cost on their hosts in the absence of selection for beneficial traits. As a result, models predict that costly CPs will be removed from the population over time by purifying selection or that CP-borne beneficial genes should be integrated into the host chromosome. However, long-term bacteria-plasmid co-culture experiments have shown that plasmids can persist for long periods, even in the absence of positive selection [87, 88]. This discrepancy between theoretical predictions and experimental observations constitutes “the plasmid paradox” [89–91]. To achieve this higher-than-predicted stability, CPs have evolved multiple mechanisms that promote their persistence at the population level. Among these mechanisms, partitioning systems and toxin-antitoxin systems are best characterized examples, which promote segregation of plasmids into daughter cells during cell division and eliminate daughter cells that fail to inherit plasmids after cell division, respectively. These systems will not be discussed further as they have been extensively reviewed elsewhere [92–96]. Instead, this review aims to focus on two mechanisms by which CPs employ to enhance their evolutionary fitness by manipulating subsequent conjugation events in their new hosts: entry exclusion and fertility inhibition.

### Entry exclusion

**Fig. 2:**
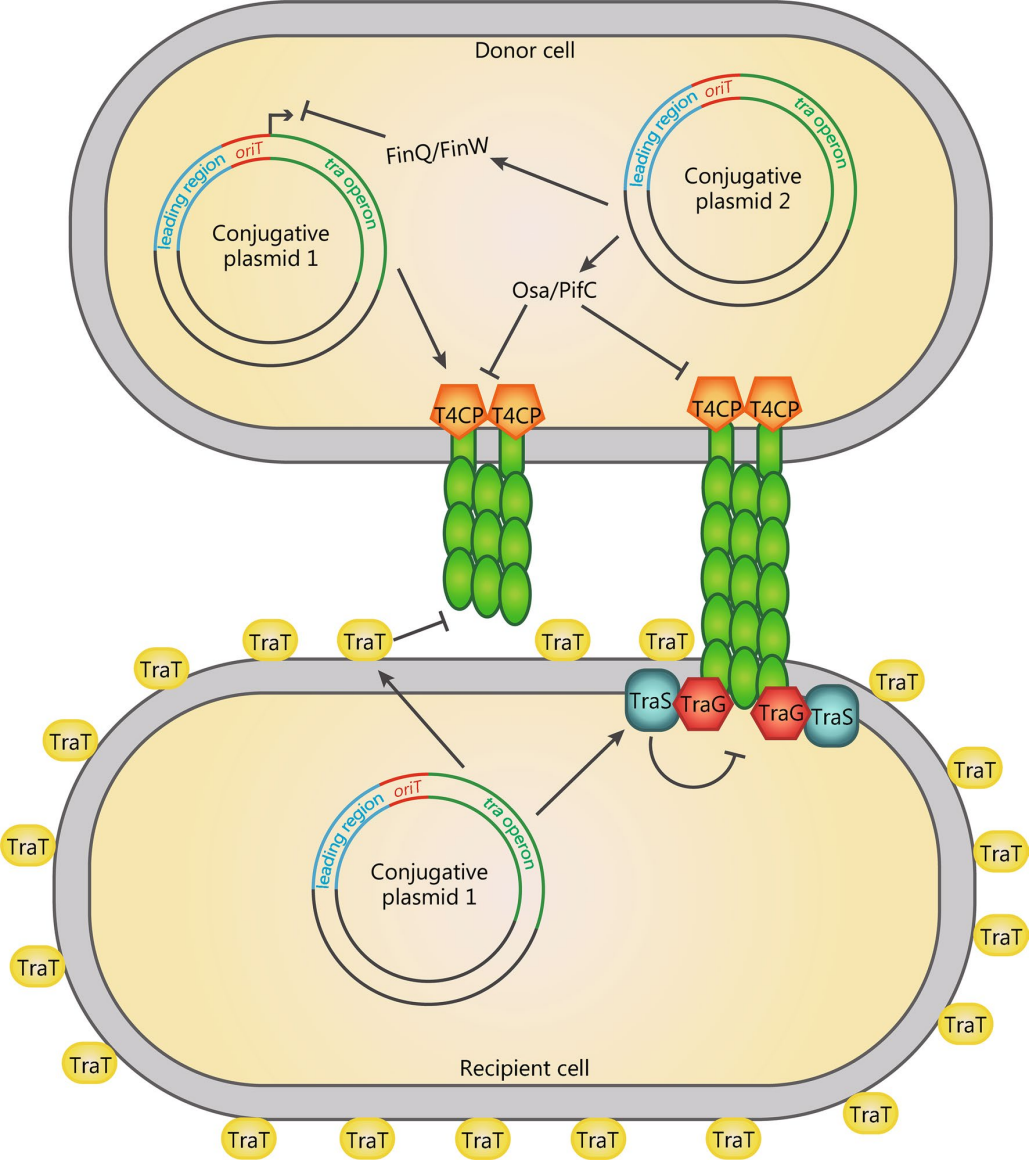
Conjugative plasmids undergo intra- and inter-incompatibility group competitions via entry exclusion and fertility inhibition. The generic plasmid CP2 prevents the conjugal transfer of CP1 from a different incompatibility group by downregulating the expression of CP1 *tra* genes and targeting the type IV coupling protein of CP1. A recipient with CP1 or CP1-related CPs that have similar entry exclusion systems such will block the entry of CP1 by destabilizing mating pair formation. CP conjugative plasmid, T4CP type IV coupling protein

Entry exclusion refers to the formation of a barrier to the horizontal transfer of CPs between bacteria carrying closely related conjugation machineries. Entry exclusion is an essential feature of CPs, and bacterial cells harboring these plasmids become poor recipients during further rounds of conjugation (Fig. 2) [97, 98]. For the F plasmid, this property is conferred by the entry exclusion locus (*traST*) at the distal end of the *tra* operon [99]. TraT is highly expressed on the outer membrane and acts to destabilize mating aggregates [100]. TraS, an inner membrane protein, interacts with TraG, a protein crucial for mating pair stabilization [101]. TraG synthesized in the donor cell was suggested to be translocated into the recipient and become quenched by TraS, resulting in blocked transfer of the T-strand [102]. The precise mechanisms of action of these exclusion factors remain incompletely understood.

The physiological importance of entry exclusion is highlighted by the failure to isolate F plasmids carrying *traS* and *traT* mutations by chemical mutagenesis [103]. F plasmid mutants which are proficient for transfer but exclusion-deficient are unstable under normal growth conditions, suggesting that entry exclusion can be essential for the stability of a CP. Additionally, excessive rounds of conjugation lead to bacterial death, due to the cell permeability induced by extensive damage in the cell membrane of the recipient [104]. This could be significantly inhibited by entry exclusion, which prevents conjugation and helps to establish immunity to cell damage.

### Fertility inhibition

To increase the probability of their long-term evolutionary success, CPs need to minimize the fitness costs they impose on their host resulting from unnecessary expression of the conjugation machinery, which expends a significant amount of cellular resources and renders the host bacterium vulnerable to predation by sex-pili targeting phages [105]. Fertility inhibition (FI) systems are ubiquitously present on CPs belonging to different incompatibility groups and inhibit the conjugal transfer of CPs [106–108]. The targets of FI systems can vary from CPs that encode the FI systems themselves to other CPs co-resident in the same bacterium [106]. Although the mechanisms by which FI systems block conjugation are poorly understood, current knowledge already shows that different FI systems target different steps of the conjugation process (Fig. 2) [106, 107].

First, as exemplified by the FinOP system, FI systems can impair the expression of genes encoding MPF systems at multiple levels. For instance, the FinQ and FinW systems inhibit transfer of the F plasmid by targeting transcription of F plasmid-encoded T4SS. FinW, encoded by IncFI plasmids such as R455, represses transcription of TraM [109], an essential component of the F plasmid relaxosome [110, 111]. On the other hand, FinQ, encoded by IncI1 plasmids such as R820a, terminates transcription of the *tra* operon at multiple sites in a Rho-independent fashion [112]. Consistent with these FI systems targeting transcription of transfer genes, long-term bacteria-plasmid co-culture experiments demonstrated that down-regulation of transfer genes expression could ameliorate the cost-of-carriage of CPs [91].

FI systems can also function without regulating expression of the conjugative machinery. Osa, encoded by the IncW plasmid pSa, inhibits the delivery of oncogenic T-DNA encoded by the Ti plasmid of *Agrobacterium tumefaciens (A. tumefaciens)* to plant cells [113]. Osa contains a ParB/Sulfiredoxin fold with both ATPase and DNase activity, with the DNase activity regulated by ATP [114]. Notably, using a partially reconstituted *A. tumefaciens* T4SS in vitro, Osa can degrade T-DNA in complex with relaxase before its translocation [115]. Osa from IncW plasmids inhibits conjugation of IncP plasmids by targeting the type IV coupling protein (T4CP), which directs the T-strand: relaxosome complex to the T4SS machinery [114]. In addition to inhibition mediated by Osa, the T4CP seems to be a major checkpoint targeted by FI systems. For instance, FinC, encoded by the mobilizable but non-conjugative plasmid CloDF13, inhibits F plasmid transfer by targeting TraD, the F plasmid T4CP, and this inhibition can be alleviated by *traD* over-expression [116]. Furthermore, PifC from IncFI or IncI1 plasmids can prevent conjugation of IncW plasmids by T4CP through unknown mechanisms [114].

## Conclusions

An arms race between CPs and bacterial defense against parasitism has been raging throughout their evolutionary history. A thorough understanding of this topic is of great clinical importance given the impeding AMR crisis as it potentially allows the prediction of future spread of CPs and can be deployed to devise interventions to combat the emergence of resistance. Although a substantial amount of research on CP-host interactions has been undertaken in the last few decades, many questions remain unanswered, and several challenges lie ahead. Firstly, most studies investigating the molecular mechanisms of CP-host interactions have focused on model plasmids such as the F plasmid in *E. coli*; studies on more clinically relevant organisms and their CPs, such as *K. pneumoniae*, *A. baumannii*, and *P. aeruginosa*, are needed [117–120]. Moreover, whether “the plasmid paradox” also holds true for clinically relevant AMR-harboring CPs and their bacterial hosts remains unknown and the answer to this question is of great medical importance as this could profoundly influence the outcomes of current measures fighting AMR such as antibiotic stewardship. Those knowledge gaps are largely due to the challenge in genetic manipulation of clinical isolates and require the development of genetic tools for several important pathogens. Another major limitation is that conjugation has mostly been examined in highly artificial environments within the laboratory, while conjugation, as a contact-dependent process, takes place within the dense and heterogeneous bacterial communities in a wide range of environments, particularly in the mammalian gastrointestinal tract. Thus, characterization of conjugation and the factors affecting its efficiency should be performed in more clinically relevant environments such as in the gastrointestinal tract and in biofilms on medical devices [121–123]. Furthermore, the leading region, as discussed above, encodes pro-colonization proteins crucial for thwarting host defense during the early stages of conjugation. However, functions of many genes residing in this region from clinically important CPs remain unstudied and warrant further investigation.

## Data Availability

Not applicable.

## Abbreviations

*A. baumannii*: *Acinetobacter baumannii*
*A. tumefaciens*: *Agrobacterium tumefaciens*
AMR: Antimicrobial resistance
Cas: CRIPSR-associated
CPs: Conjugative plasmids
*E. coli*: *Escherichia coli*
CRISPR: Clustered regularly interspaced palindromic repeats
HGT: Horizontal gene transfer
*K. pneumoniae*: *Klebsiella pneumoniae*
MGEs: Mobile genetic elements
MPF: Mating pair formation
MTase: Methyltransferase
NMR: Nuclear magnetic resonance
OB: Oligosaccharide-binding
*P. aeruginosa*: *Pseudomonas aeruginosa*
*P. putida*: *Pseudomonas putida*
REase: Restriction endonuclease
R-M: Restriction-modification
SMRT: Single-molecule real-time
ssDNA: Single-stranded DNA
SSB: Single-stranded DNA binding
SSB-Ct: C-terminal tail of ssDNA binding proteins
T4CP: Type IV coupling protein
T4SS: Type IV secretion system
